# Numerical Analysis of Microfluidic Motors Actuated by Reconfigurable Induced-Charge Electro-Osmotic Whirling Flow

**DOI:** 10.3390/mi16080895

**Published:** 2025-07-31

**Authors:** Jishun Shi, Zhipeng Song, Xiaoming Chen, Ziang Bai, Jialin Yu, Qihang Ye, Zipeng Yang, Jianru Qiao, Shuhua Ma, Kailiang Zhang

**Affiliations:** 1 School of Control Engineering, Northeastern University at Qinhuangdao, Qinhuangdao 066004, China; 2 Hebei Key Laboratory of Micro-Nano Precision Optical Sensing and Measurement Technology, Qinhuangdao 066004, China; 3 College of Mechanical and Electrical Engineering, Northeast Forestry University, Harbin 150040, China

**Keywords:** induced-charge electro-osmosis, microfluidic motors, protein detection, whirling flow

## Abstract

The detection of proteins plays a key role in disease diagnosis and drug development. For this, we numerically investigated a novel microfluidic motor actuated by an induced-charge electro-osmotic (ICEO) whirling flow. An alternating current–flow field effect transistor is engineered to modulate the profiles of ICEO streaming to stimulate and adjust the whirling flow in the circle microfluidic chamber. Based on this, we studied the distribution of an ICEO whirling flow in the detection chamber by tuning the fixed potential on the gate electrodes by the simulations. Then, we established a fluid–structure interaction model to explore the influence of blade structure parameters on the rotation performance of microfluidic motors. In addition, we investigated the rotation dependence of microfluidic motors on the potential drop between two driving electrodes and fixed potential on the gate electrodes. Next, we numerically explored the capability of these microfluidic motors for the detection of low-abundance proteins. Finally, we studied the regulating effect of potential drops between the driving electrodes on the detection performance of microfluidic motors by numerical simulations. Microfluidic motors actuated by an ICEO whirling flow hold good potential in environmental monitoring and disease diagnosis for the outstanding advantages of flexible controllability, a simple structure, and gentle work condition.

## 1. Introduction

The detection of low-abundance proteins plays a significant role in the prognosis, diagnosis and therapy of many diseases, such as breast cancer, diabetes mellitus, and so on, because there are numerous protein cancer biomarkers in the tissues or body fluids of patients. If the species and concentrations of these proteins are detected, we can achieve the diagnosis, monitoring, and prediction of the disease progression [1]. For this, many approaches have been developed to detect the low-abundance proteins, including Western blotting, gel electrophoresis, mass spectrometry, and an enzyme-linked immunosorbent assay [2]. It is worth noting that the above methods may take considerable time and effort to realize the effective detection of low-abundance proteins. Microfluidics-based approaches can economically realize the detection of small-volume protein samples without cross contaminations in a short time, holding promising potential in the detection of low-abundance proteins [3].

Diverse mechanisms have been used to develop microfluidic devices for the detection of proteins, involving optical fields, electrochemistry, mechanical sensing, mass spectrometry, and biomolecule recognition [4]. Optical approaches can detect protein concentrations, species, and conformations based on the changes caused by the interaction between light and proteins [5]. Fluorescence approaches can achieve high-sensitivity quantification with fluorescein (FITC) or quantum dot labeling, but such methods heavily rely on complex labeling procedures and optical filtering systems [6,7]. Although methods of SPR present an excellent performance in the real-time monitoring of protein binding events by using the refractive index change on the surface of metal films in a label-free fashion, these approaches presented limitations in their deep integration with microfluidic chips for a bulky optical path system [8,9]. Localized surface plasmon resonance (LSPR) significantly strengthens sensitivity and simplifies the instrument through the localized light field enhancement effect of nanostructures [10]. The electromagnetic field enhancement effect of precious metal nanoparticles aims to achieve the detection of SERS at the level of a single molecule, but the unsatisfactory controllability of nano-substrate preparation and poor signal repeatability are still bottlenecks for the applications [11,12,13]. In addition, the colorimetric methods based on the color development reaction have the advantages of a low cost and good readability, but the sensitivity is insufficient in the detection of low-abundance proteins [14,15]. Electrochemical approaches can realize the detection of proteins in simple devices with a fast response, which mainly include amperometry, impedance spectroscopy, and field effect transistors [5,16,17]. The enzyme-labeled probe catalyzes the substrate to produce electroactive substances and then detects the redox current, but the selectivity is easily interfered by impurities [18,19,20]. Impedance spectroscopy can identify protein binding through small changes in the double-layer capacitance or charge transfer resistance for dynamic monitoring, but the sensitivity of such methods is difficult to promote for tricky low-frequency noise [21,22]. A field effect transistor (FET) is a good tool to extend the detection range of electrochemical methods [23]. With the help of the ultra-high carrier mobility and surface charge sensitivity of two-dimensional materials, such as graphene and molybdenum disulfide, the limit of detection can reach the femtomolar level [24]. By combining microfluidic chips with electrospray ionization mass spectrometry, the identification of protein species in complex samples can be achieved. This technology lies in the accurate determination of the molecular weight and the analysis of post-translational modifications, but heavily depends on large instruments [25,26,27,28]. Mechanical sensors, such as microcantilever beams [29,30] and surface acoustic wave sensors [31,32,33,34,35], can achieve label-free and multi-channel detection by detecting physical structural deformation and motion characteristics caused by protein bonding. The complex signal analysis of mechanical sensors may affect the performance of mechanical sensors in some applications. In addition, mechanical sensors are sensitive to environmental interference. The microfluidic enzyme-linked immunosorbent assay (ELISA) system and aptamer technology use single-molecule counting technology to improve the sensitivity to the level of fg/mL, but antibody stability and cross-reaction control still need to be optimized [36,37,38,39]. Molecular imprinting polymer (MIP) technology constructs artificial recognition sites through a template method and has shown potential in high-temperature and harsh environment detection, but the imprinting efficiency and template elution effect still restrict the sensitivity [40,41].

Microfluidic motors can convert various energies into mechanical energy for desired mechanical movement, which exhibit incredible potential in addressing tricky tasks in the field of drug delivery, analytical sensing, energy generation, and assisted fertilization. Noticeably, rotational microfluidic motors can adsorb proteins in the solution repeatedly to significantly improve the detection, which is a promising alternative in the detection of proteins. In this scenario, it is the prerequisite to find gentle and effective methods to actuate microfluidic motors. Various mechanisms have been investigated to achieve the actuation of microfluidic motors, mainly including chemical energy, light, ultrasound, and magnetic fields. Although microfluidic motors driven by chemical energy present a good motion performance, their applications may be strictly constrained by limited working time. Despite light and ultrasound energies being able address biomedical and environmental issues effectively, these techniques may suffer from low penetrability and a serious intensity decrease when penetrating the obstacles. Magnetic fields are a promising propulsion strategy, but motors may have high requirements in the fabrication of the materials. Therefore, it is necessary to seek a novel method to drive microfluidic motors for the detection of low-abundance proteins.

Under the action of an AC electric field, the charges in the diffuse layer migrate along or against the electric field lines. Consequently, induced-charge electro-osmotic (ICEO) streaming can be generated on the gate electrode. The profile and magnitude of ICEO streaming can be adjusted flexibly by reshaping bipolar electrodes, redistributing the driving electrodes, and modulating the potential on the bipolar electrodes. Among these approaches, tuning the fixed potential on the gate electrode may be a flexible and easy-operation method to reconfigure the profile of ICEO streaming. Interestingly, when we array the electrodes around the center of a circle chamber, an ICEO whirling flow can be generated and modulated by adjusting the working parameters. An ICEO whirling flow can actuate microfluidic motors in a gentle and flexible fashion. Therefore, an ICEO whirling flow holds excellent potential in the actuation of microfluidic motors for the detection of low-abundance proteins.

Encouraged by the above aspects, we put forward a novel microfluidic motor driven by an ICEO whirling flow for the detection of low-abundance proteins. Firstly, we investigated the modulation effect of an alternating current–flow field effect transistor (AC-FFET) on the ICEO whirling flow by simulations. Moreover, the ICEO whirling flow was engineered in the actuation of microfluidic motors, and the parametric influences of the blade structure and voltage on their rotation performances were explored numerically. Furthermore, we validated the feasibility of microfluidic motors in the detection of proteins with various concentrations by numerical simulations. Finally, we conducted simulations to study the regulating effect of potential drops between driving electrodes on the detection of proteins. These microfluidic motors hold good potential in environmental monitoring and disease diagnosis for the merits of a low cost and gentle condition.

## 2. Results of Simulation Model of Microfluidic Motors Actuated by ICEO Whirling Flow

### 2.1. Design of Microfluidic Motors

**Figure 1:**
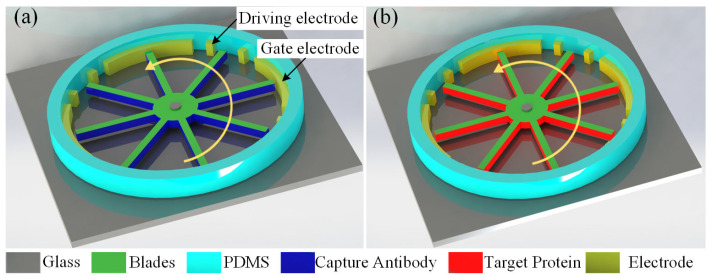
Schematics of microfluidic motors powered by ICEO whirling flow for detection of proteins. (**a**) Configuration of microfluidic motors by ICEO whirling flow. (**b**) Diagram of protein detection with microfluidic motors.

This work designed a microfluidic motor actuated by a reconfigurable ICEO whirling flow and the schematic diagram of the microfluidic motors is presented in Figure 1a. The microfluidic motors mainly consist of a circular chamber, a blade, and electrodes. The electrodes are distributed on the inner wall of the circle chamber. The blade is assembled on the stator, which is located in the middle of the circle chamber. We can fabricate the mold of the circle chamber, pour PDMS, and conduct a curing treatment to obtain the circle chamber of the micromotor. The electrode structure can be prepared with liquid metal. Liquid metal is poured in the mold of the designed electrodes at a temperature above the melting point and solidified at temperatures below the melting point to obtain the electrodes. The blade can be fabricated with two-photon 3D printing technology. Optical tweezers can be used to assemble the electrodes and blades in the circular chamber. Based on the above design and fabrication, we can obtain microfluidic motors for the detection of proteins.

An AC signal was employed on the left driving electrodes and the right one was grounded to create the ICEO streaming on the gate electrodes. A fixed potential was applied on the gate electrode to modulate the profile of the ICEO streaming. The widths of the left and right driving and gate electrodes were 50, 50, and 250 μm, respectively. Four sets of electrodes were designed to stimulate the whirling flow in the detection chamber with a diameter of 250 μm. An inlet was designed at one side of the detection chamber for the injection of the solutions containing proteins, and the outlet was designed at the opposite side for the collection of the waste samples. Under the action of the electric field, ICEO streaming can be stimulated on the gate electrodes. As a result, the ICEO whirling flow under the combined action of four pairs of ICEO streaming can be created to actuate microfluidic motors. Moreover, the rotation velocity of microfluidic motors can be flexibly tuned by changing the potential drops between the driving electrodes and the fixed potential on the gate electrodes. Blades of microfluidic motors can enlarge the reaction area and promote the detection efficiency by increasing the possibility of bonding proteins. After successive rotation, low-abundance proteins can be detected.

### 2.2. Computational Model

#### 2.2.1. AC-FFET-Modulated ICEO Whirling Flow 

To configure the ICEO whirling flow, the left driving electrode employed an AC signal $\emptyset =A_{1}cos(\omega_{1}t)$, the right driving electrode was grounded ∅=0, and the middle gate electrode applied a fixed potential $\emptyset =A_{2}cos(\omega_{2}t)$. It was assumed that the solution had uniform conductivity and thus the potential in the detection chamber can be calculated by the Laplace equation [42,43].

(1) $$\nabla \emptyset^{2}=0$$

With the action of an electric field E=−∇∅, an electric double layer (EDL) can be created on the surface of the middle gate electrode. In this scenario, the system consisting of an electrolyte and electrode can be equivalent to an RC circuit. After the RC relaxation time, the EDL is charged fully and gives rise to a zeta potential drop. When no fixed potential is employed in the middle gate electrode for the diffuse layer, the induced Zeta potential is [42,43]:

(2) $$\xi_{fl}\left(t\right)=\emptyset_{OHP}-\emptyset \left(t\right)=\frac{1}{1+\delta }\left[\frac{A_{1}}{2}\cos\left(\omega t\right)-\emptyset \left(t\right)\right]=\frac{1}{1+\delta }Ex\cos\left(\omega t\right),$$

where $\emptyset_{OHP}$ and $\emptyset \left(t\right)$ indicate the transient potential at the outer Helmholtz plane and in the bulk, and δ stands for the surface physical capacitance ratio of the diffuse layer $C_{d}=\varepsilon_{f}/\lambda_{D}$ and the stern layer $C_{S}$. When the gate electrode has a fixed potential $\emptyset =A_{2}cos(\omega_{2}t)$, the charge–voltage relationship in the diffuse layer is [42,43]:

(3) $$\xi_{fi}\left(t\right)=\frac{1}{1+\delta }\left[A_{2}\cos\left(\omega t\right)-\frac{A_{1}}{2}\cos\left(\omega t\right)+\frac{A_{1}}{2}\cos\left(\omega t\right)-\emptyset \left(t\right)\right]\begin{array}{c} =\frac{1}{1+\delta }\left(Ex+A_{2}-\frac{A_{1}}{2}\right) \end{array}$$

Under such circumstances, the surface charge density in the EDL is [42,43]:

(4) $$q=C_{d}\xi_{fi}\left(t\right)=\frac{C_{d}}{1+\delta }\left(Ex+A_{2}-\frac{A_{1}}{2}\right)\cos\left(\omega t\right),$$

Based on the Helmholtz–Smoluchowski formula, we can derive the transient slip velocity on the polarized gate electrode [42,43].

(5) $${u_{slip}=-\frac{\varepsilon_{f}}{\eta }\xi }_{fi}\left(t\right)E_{t}=-\frac{\varepsilon_{f}E_{t}}{\eta \left(1+\delta \right)}\left(Ex+A_{2}-\frac{A_{1}}{2}\right)\cos\left(\omega t\right),$$

The time-average slip velocity is [42,43]:

(6) $${<u}_{slip}>=-\frac{\varepsilon_{f}E}{2\eta \left(1+\delta \right)}\left(Ex+A_{2}-\frac{A_{1}}{2}\right),$$

The slip velocity caused by the ICEO effect is applied on the gate electrodes. Under the combined actuation of multiple ICEO streaming, a whirling flow is formed in the detection chamber. By substituting Equation (6) into the Navier–Stokes equation, we can calculate the flow field.

(7) $$\rho \left(u\cdot \nabla \right)u=\nabla \left[-pI+K\right]+F,$$

(8) $$\nabla \left(\rho u\right)=0,$$

(9) $$K=\mu \left(\nabla u+\nabla {\left(\nabla u\right)}^{T}\right)-\frac{2}{3}\mu \left(\nabla u\right)I,$$

The protein samples are injected into the chamber; thus, a flow rate is employed on the inlet.

(10) $$v=v_{0}n,$$

Meanwhile, the pressure at the outlet is set as:

(11) $$p=p_{0},$$

#### 2.2.2. Rotation of Microfluidic Motors

Microfluidic motors can rotate around the center of the detection chamber, which is the center of mass of the blades.

(12) $$u_{c}=u+\left(R-I\right)\left(X_{c}-X_{M}\right)$$

(13) $$X_{c}=\frac{\int X\;dL}{\int dL}$$

(14) $$X_{M}=\frac{\int \rho Xd\;dA}{m}$$

where $u_{c}$ is the source displacement at the center of the rotation center, $u_{c,dest}$ stands for the destination displacement at the center of joint, $X_{c}$ is global coordinates of the center of the rotation center, $X_{M}$ is the center of mass, and I is an inertia moment of the microfluidic motors. Microfluidic motors are defined as the rigid region. Under the applied force and moment, microfluidic motors can rotate around the rotation center.

(15) $$m\frac{d^{2}}{dt^{2}}\left(u+\left(R-E_{3}\right)\left(X_{M}-X_{c}\right)\right)+\sum F_{I}=\sum F_{ext}$$

(16) $$I_{Z}\frac{d^{2}\emptyset }{dt^{2}}+\sum M_{I}=\sum M_{ext}$$

(17) $$I_{Z}=\int \left(\left(X-X_{M}\right)\cdot \left(X-X_{M}\right)\right)\rho d\;dA$$

where m=∫ρd dA is the mass of the microfluidic motors and $I_{Z}$ indicates the inertia moment of the microfluidic motors.

### 2.3. Detection of Low-Abundance Proteins

Based on the fluid–structure interaction, we can calculate the distribution of the flow field by considering the actuation of the microfluidic motors. We use the standard convention–diffusion equation to describe the concentration of the proteins.

(18) $$\frac{\partial c_{i}}{\partial t}+\nabla \cdot J_{i}+u\cdot {\nabla c}_{i}=R_{i}$$

(19) $$J_{i}=-D_{i}{\nabla c}_{i}$$

where $D_{i}$ and $c_{i}$ indicate the thermal diffusivity and concentrations of the protein samples.

To characterize the detection of the proteins, the flux condition on the blades is set as:

(20) $${-n\cdot J}_{i}=J_{0,cp}$$

We also applied the concentration condition of the proteins at the inlet and the outlet was set as the no-flux boundaries.

(21) $$c_{i}=c_{0,i}$$

(22) $${-n\cdot J}_{i}=0$$

Due to the low concentration of proteins, we assume that the samples have no influence on the detection solution. Based on this, we can use the advection–diffusion reaction equation to describe the distribution of the protein concentration in the chamber during the detection process.

(23) $$\frac{\partial c_{s,j}}{\partial t}+\nabla_{t}\cdot \left(uc_{s,j}\right)+\nabla_{t}\cdot \left(-D_{j}{\nabla_{t}c}_{s,j}\right)=R_{s,j}$$

(24) $$N_{s,j}=-D_{j}{\nabla_{t}c}_{s,j}$$

(25) $$\frac{\partial c_{b,j}}{\partial t}=R_{b,j}$$

where u indicates the flow rate, $c_{s,j}$ and $D_{j}$ stand for the concentration and diffusion coefficient of the proteins, and $R_{s,j}$ is the rate of the chemical reaction. The second and third terms on the left side are the advection and diffusion effects giving rise to the concentration of proteins.

## 3. Simulation Analysis of Microfluidic Motor Rotation Actuated by ICEO Whirling Flow

### 3.1. Modulation Effect of AC-FFET on ICEO Streaming for the Formation of Whirling Flow

**Figure 2:**
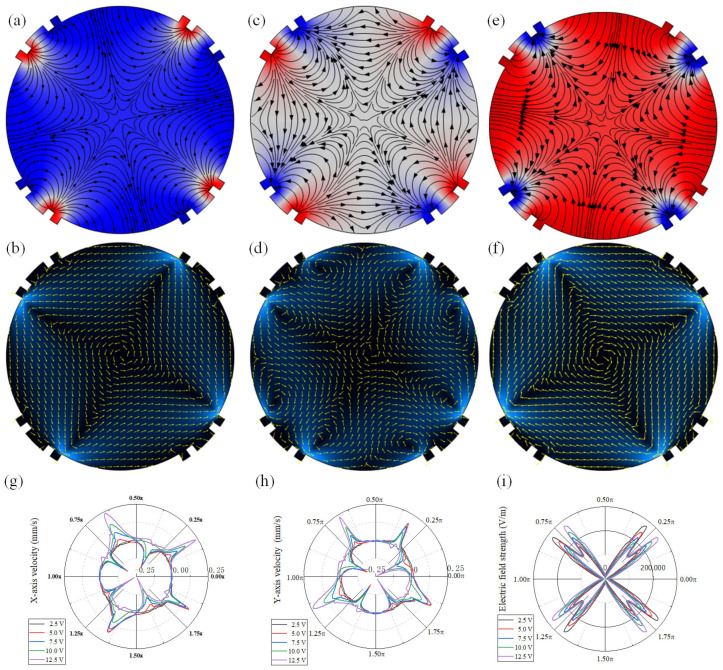
Modulation effect of fixed potential on the flow field. (**a**–**f**) Distribution of electric and flow field under different voltage parameters: (**a**,**b**) *A*_1_ = 15 V, *A*_2_ = 7.5 V, and *f* = 200 Hz; (**c**,**d**) *A*_1_ = 15 V, *A*_2_ = 3 V, and *f* = 200 Hz; and (**e**,**f**) *A*_1_ = 15 V, *A*_2_ = 13 V, and *f* = 200 Hz. (**g**,**h**) Effect of fixed potential on the *u*_x_/*u*_y_. (**i**) Influence of fixed potential on the electric field strength.

We first investigated the modulation performance of AC-FFET on the distribution of the electric and flow field in the chamber by regulating the fixed potential on gate electrodes, when an AC signal of *A*_1_ = 15 V and *f* = 200 Hz was employed on the driving electrodes. The electric field distribution at *A*_2_ = 7.5 V is given in Figure 2a. Four pairs of symmetrical ICEO streaming were formed on the gate electrodes with the action of an electric field, and no whirling flow was generated (Figure 2b). By decreasing *A*_2_ to 3 V, an anticlockwise whirling flow was created under the action of four symmetrical ICEO streaming (Figure 2c,d). Interestingly, when increasing *A*_2_ to 13 V, the electric field was reshaped (Figure 2e) and a clockwise whirling flow was created (Figure 2f). Furthermore, we quantitatively investigated the modulation effect of the fixed potential on the flow velocity *u*_x_ and *u*_y_, as given in Figure 2g, h. With the rising fixed potential from 2.5 V to 12.5 V, the directions of *u*_x_ and *u*_y_ were tuned gradually. Moreover, the amplitudes of the flow rate were enhanced slightly. Under such conditions, the distribution of the electric field strength is given in Figure 2i. Therefore, the whirling flow can be formed under the combined action of four pairs of ICEO streaming, and the direction and amplitude can be tuned by adjusting the potential drops between the driving electrodes and fixed potential on the gate electrodes.

### 3.2. Influence of Blade Numbers on the Performance of Microfluidic Motors

**Figure 3:**
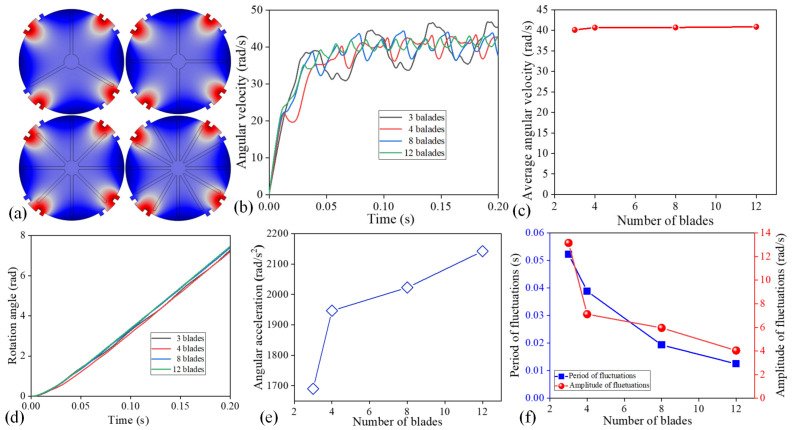
The effect of blade number on the performance of microfluidic motors. (**a**) Simulation model of microfluidic motors with different blade numbers. (**b**) Angular velocity of microfluidic motors with different blades versus time. (**c**) Influence of blade number on the angular acceleration in the startup stage. (**d**) Rotation angle of microfluidic motors with various blades. (**e**) The influence of blade numbers on the angular acceleration. (**f**) The fluctuation period and amplitude of microfluidic motors with blades of various numbers.

To study the influence of blade numbers on the performance of microfluidic motors, the blade number was set as 3, 4, 8, and 12 at the length of 260 μm (Figure 3a). As time increased, the angular velocity of microfluidic motors with diverse blade numbers showed a similar change trend (Figure 3b). The angular velocity of the microfluidic motors showed a significant increase at *t* = 0~0.05 s. In this scenario, the velocity of the microfluidic motor blades was smaller than the flow rate and the microfluidic motors were successively accelerated by the ICEO whirling flow. With the consistent actuation of the ICEO whirling flow, the angular velocities of the microfluidic motors were stabilized at about 40 rad/s with periodic fluctuations (Figure 3c). The rotation fluctuation period and amplitude of the microfluidic motors decreased with the rising blade number. The rotation angle of the microfluidic motors with different numbers of blades presented the same trend over time and reached 7.3 rad within 0.2 s (Figure 3d). When the number of blade microfluidic motors was increased from three to four, the angular acceleration was rapidly increased from 1689.79 to 1946.63 rad/s^2^ (Figure 3e). When the blade number was increased from four to eight, the angular acceleration began to slow down. With further increasing the number of blades to 8 and 12, the angular acceleration was increased to 2022.60 and 2142.40 rad/s^2^. We also studied the effect of the number of blades on the rotation fluctuation of the microfluidic motors in the stable rotation stage. The periodic rotation fluctuation was caused by the uneven distribution of the flow field. The fluctuation amplitudes were reduced from 13.15 to 4.04 rad/s and the fluctuation periods were decreased from 0.052 to 0.012 s with the rising blade number from 3 to 12 (Figure 3f). Therefore, the microfluidic motors with many blades needed less time to reach stable rotation with the short fluctuation period and the small fluctuation amplitude.

### 3.3. Effect of Blade Length on the Rotation Performance of Microfluidic Motors

**Figure 4:**
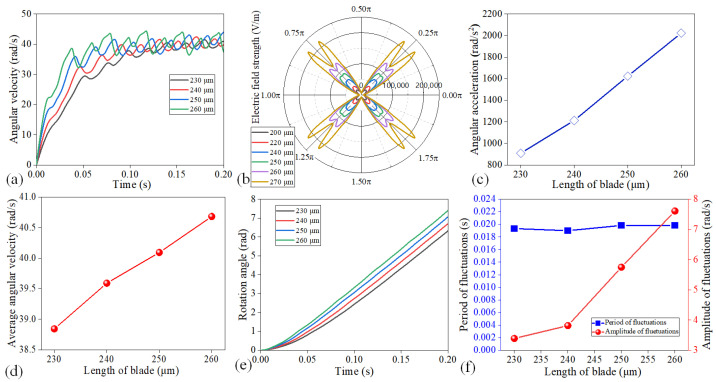
The effect of blade length on the rotation of microfluidic motors. (**a**) The effect of blade length on the angular velocity. (**b**) Electric field strength along the different-sized circles. (**c**) The effect of blade length on angular acceleration of microfluidic motors at the startup stage. (**d**) The influence of blade length on the average angular velocity of microfluidic motors after stable rotation. (**e**) Rotation angle of microfluidic motors with different blade lengths. (**f**) The effect of blade length on the speed fluctuation of microfluidic motors.

We then studied the effect of the blade length on the rotation of the microfluidic motors. The angular velocities of the eight-blade microfluidic motors with diverse blade lengths are shown in Figure 4a. Microfluidic motors with different blade lengths can be actuated rapidly and then reach a dynamic stable state with diverse rotation angular velocities. The angular velocities of the microfluidic motors exhibited distinct growth trends and fluctuations caused by the decreasing flow rate of the whirling flow radially inward. The electric field strength presented a downward trend with the decreasing diameter of the circles (Figure 4b). Microfluidic motors with long blades experienced a strong driving force of the ICEO whirling flow and obtained a high stable rotation speed. The relationship between the angular acceleration and the blade length in the startup stage is presented in Figure 4c. The average accelerations of the microfluidic motors were increased from 909.38 to 2022.60 rad/s^2^ at *t* = 0.02 s with increasing the blade length from 230 μm to 260 μm. The angular acceleration showed an upward trend with the increasing length of the blade because long blades can provide sufficient actuation force in the startup stage. Moreover, the average angular velocity at the stable rotation exhibited an upward trend from 38.84 to 40.68 rad/s with the increase in the blade length from 230 to 260 μm (Figure 4d). Microfluidic motors with long blades needed less time to reach stable rotation with a high rotation velocity. By increasing the blade lengths from 230 to 260 μm, the rotation angle within 2 s was increased from to 6.325 to 7.397 rad (Figure 4e). The fluctuation period remained around 0.019 s with the increasing blade length (Figure 4f). The fluctuation amplitude showed an evident increasing trend from 3.39 to 7.61 rad/s with the increase in the blade length from 230 to 260 μm (Figure 4f). Therefore, long blades can enhance the angular acceleration and stable angular velocity at the startup stage, but they cause a fluctuation of microfluidic motors.

### 3.4. Influence of Electric Field on the Rotation Performance of Microfluidic Motors

**Figure 5:**
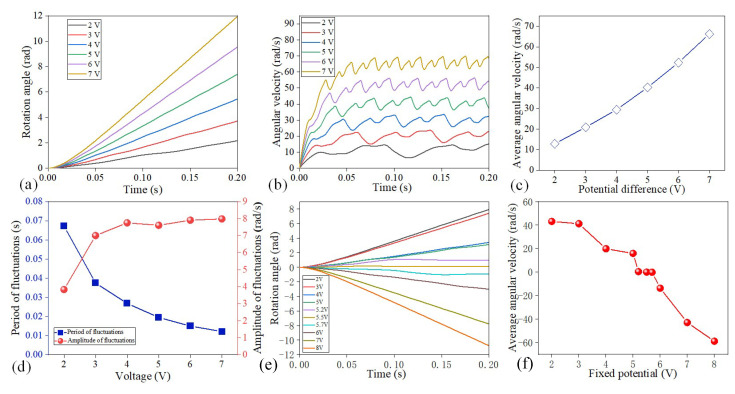
Rotation of microfluidic motors under diverse electric fields. (**a**) Rotation of microfluidic motors under various potential drops. (**b**) Influence of potential drop on the angular velocity of microfluidic motors. (**c**) Average angular velocity of microfluidic motors after stabilization under various potential drops. (**d**) Fluctuation period and amplitude under various potential drops. (**e**) Influence of fixed voltage on the rotation angle of microfluidic motors. (**f**) Effect of fixed voltage on angular velocity after stable rotation.

The distribution of the electric field plays a critical role in the rotation of microfluidic motors. For this, we first studied the effect of the voltage drop between the driving electrodes on the rotation performance of microfluidic motors under the fixed potential of 3 V on the gate electrode. The rotation angle of the microfluidic motors under the potential drop of 2~7 V are presented in Figure 5a. Although the rotation angle presented a similar increasing trend under different voltages over time, the microfluidic motors rotated faster under a high potential drop. At *t* = 0.2 s, by increasing the potential drop from 2 to 7 V, the rotation angle of the microfluidic motors presented a rising trend from 2.17 to 11.96 rad. The angular velocity of the microfluidic motors over time under various potential drops is shown in Figure 5b. The angular velocity increased rapidly at the beginning and then exhibited different growth trends and fluctuations under different potential drops. With increasing the potential drop from 2 to 7 V, the average angular velocity exhibited an increasing trend from 12.84 to 66.28 rad/s (Figure 5c). The fluctuation periods were reduced from 0.067 to 0.012 s, when the potential drop was increased from 2 to 7 V (Figure 5d). The fluctuation amplitude initially exhibited an upward trend with the rising potential drop from 2 to 4 V, and then the amplitude tended to stabilize with the voltage exceeding 4 V (Figure 5d). Next, we studied the effect of the fixed potential on the rotation of the microfluidic motors (Figure 5e,f). At the potential drops of 5 V, the angular velocities decreased from 43.14 to −58.87 rad/s when the fixed potentials on the gate electrode were raised from 2 to 8 V. Noticeably, the rotation direction of the microfluidic motors was switched at the fixed potential of 5.5 V (Figure 5f). Therefore, we can tune the rotation performance of microfluidic motors by adjusting the potential drops between the driving electrodes and fixed potential on the gate electrodes.

## 4. Simulation Analysis of Protein Detection with Microfluidic Motors Actuated by ICEO Whirling Flow

In this section, we first verified the capability of the microfluidic motors in the detection of proteins by a numerical simulation. Moreover, the performance of the microfluidic motors in the diverse-concentration proteins was numerically explored. Based on this, we exploited the modulation effect of potential drops between driving electrodes on the detection of proteins.

### 4.1. Validation of Detection Capability of Microfluidic Motors Actuated by ICEO Whirling Flow by Numerical Simulations

**Figure 6:**
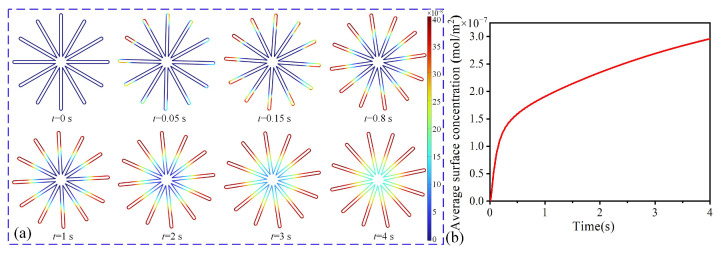
Detection performance of microfluidic motors at different times. (**a**) Adsorption of proteins on the blade surface. (**b**) Average surface concentration of proteins over time.

In this section, we numerically demonstrated the feasibility of microfluidic motors in the detection of proteins. The distribution of adsorbed proteins on the blade surfaces of microfluidic motors at different times is given in Figure 6a. There were no adsorbed proteins on the microfluidic blade surface at *t* = 0 s. As the proteins diffused in the detection chamber, the blade tips first contacted and adsorbed proteins at *t* = 0.05 s. At *t* = 0.15 s, many proteins were adsorbed on the tips of the blades. Afterwards, the multilayer of proteins was adsorbed on the blade surface of the microfluidic motors for an enhanced coloring gradient (*t* = 0.8~4 s). As the proteins steadily diffused, the absorbed proteins on the blade surfaces continued to increase. The average surface concentration of the adsorbed proteins on the blades over time is given in Figure 6b. At *t* = 0~0.3 s, the average surface concentration presented a linear rise for rapid adsorption on available active sites. At *t* = 0.3~0.6 s, the average surface concentration began to slow down and entered the deceleration stage due to site occupancy and steric hindrance.

### 4.2. Simulation Analysis of Protein Detection Under Diverse Concentrations

**Figure 7:**
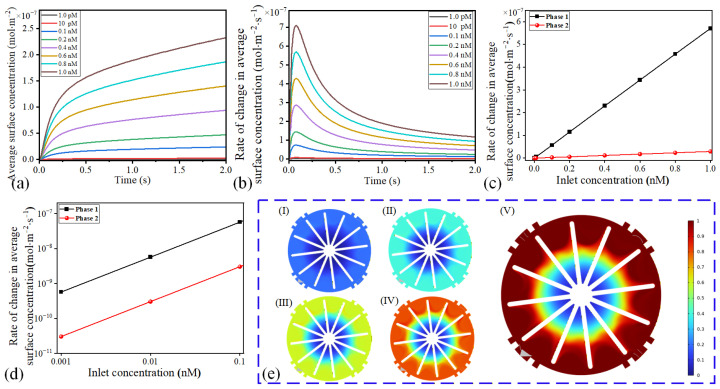
The effect of different inlet concentrations on the detection of proteins. (**a**,**b**) Average surface concentration and its change rate under different input concentrations. (**c**,**d**) The change rate of surface concentration in the start-up stage and the stable change stage at different input concentrations. (**e**) The concentration distributions under different conditions at t = 1 s, corresponding to input concentrations of 0.2, 0.4, 0.6, 0.8, and 1 nM in I–V, respectively.

We numerically investigated the performance of microfluidic motors in the detection of proteins with diverse concentrations. The average surface concentration increased rapidly at *t* = 0~0.08 s and then the increase rate gradually stabilized (Figure 7a). By increasing the concentration of proteins from 0.001 to 1.0 nM, the average surface concentrations were raised from 2.41 × 10^−10^ mol/m^2^ to 2.32 × 10^−7^ mol/m^2^ at *t* = 2 s (Figure 7a). In addition, at high initial concentrations, proteins were adsorbed on the blade surfaces at a fast velocity (Figure 7b). The change rates of the average surface concentration in the startup stage and the stable stage are given in Figure 7c,d. In the two stages, the change rate of the average surface concentration increased linearly with the increase in the protein concentration, which indicates that the microfluidic motors can effectively detect the proteins. Moreover, the concentration of proteins had a more significant effect on detection in the first stage because the growth rates of the average surface concentration were increased from 5.684 × 10^−10^ to 5.705 × 10^−7^ mol/m^2^·s. In the second stage, the change rates were increased from 3.028 × 10^−11^ to 2.932 × 10^−8^ mol/m^2^·s. The surface concentration distribution over time at the concentrations of 0.2~1 nM are presented in Figure 7e. By raising the concentration of proteins, many proteins in the solution facilitated the adsorption of proteins on the surface of the blades of the microfluidic motors. Therefore, microfluidic motors actuated by an ICEO whirling flow can realize the detection of proteins.

We also compared the performance of this microfluidic motor in the detection of proteins to that of existing methods, as given in Table 1. According to Table 1, this microfluidic motor has a lower detection limit and shorter detection time.

### 4.3. Simulation Analysis of Modulation Effect of Potential Drops on the Detection Performance of Microfluidic Motors

**Figure 8:**
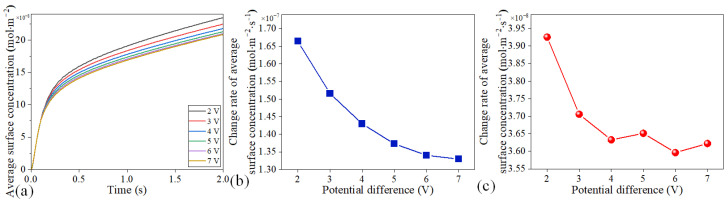
Modulation effect of potential drop on the detection of protein. (**a**) Average surface concentration over time under diverse potential drops. (**b**) Change rate of average concentration under various potential differences in the startup stage. (**c**) Change rate of average concentration under various potential differences in the stable stage.

We also studied the modulation effect of the potential drop on the detection of proteins by numerical simulations. The surface concentration over time under different potential drops is presented in Figure 8a. At *t* = 2 s, with increasing the potential drop from 2 to 7 V, the average surface concentrations on the blades were reduced from 2.3465 × 10^−7^ to 2.0854 × 10^−7^ mol/m^2^. As the potential drop increased, the growth rate of the surface concentration showed a downward trend at *t* > 0.08 s. The change rate of the surface concentration experienced a slight drop when the potential drop was increased from 5 V to 7 V because the reaction rate had slowed down, as shown in Figure 8b. After *t* = 1.8 s, the change rate of the average surface concentration was reduced from 3.9255 × 10^−8^ to 3.6223 × 10^−8^ mol/m^2^·s with the increase in the voltage from 2 to 7 V, as shown in Figure 8c. When the voltage was increased from 2 to 4 V, the surface concentration change rate gradually decreased. When the voltage was further increased to 6 V, the change rate reached the lowest value and then tended to be stable. Therefore, we can adjust the detection performance of microfluidic motors by changing the potential difference between the driving electrodes.

### 4.4. Discussion of the Possibility of Prototyping Microfluidic Motors

The processing of microfluidic motors is the key to determining whether microfluidic motors can be realized in real life. The circle chamber of microfluidic motors can be fabricated by a standard soft lithography process and this technique has been used to fabricate multifarious microfluidic structures [47]. The fabrication of liquid metal electrodes is also relatively mature, which has been reported in many papers for dielectrophoresis-based cell separation [48]. Optical tweezers are a very precise operation method of microscale objects, which can assemble the blade and electrodes in the circle chamber [49]. Therefore, the prototype of this microfluidic micromotor is possible in real life.

## 5. Conclusions

Herein, we numerically investigated a novel microfluidic motor actuated by an ICEO whirling flow for the detection of low-concentration proteins. AC-FFET was engineered to modulate the direction and amplitude of the ICEO whirling flow. Based on this, we established a fluid–structure interaction computational model and explored the influence of the blade number, length, potential drops, and fixed potential on the rotation performance of the microfluidic motors. Many blades can facilitate microfluidic motors to reach a stable rotation with the short fluctuation period and weak fluctuation amplitude within a short time. The angular acceleration and stable angular velocity can be strengthened by increasing the blade length at the startup stage, but the fluctuation of the microfluidic motors became more evident. The rotation speed of the microfluidic motors can be enhanced by increasing the potential drop between the driving electrodes, and the rotation direction of the microfluidic motors can be switched by tuning the fixed potential to the gate electrode. Moreover, we numerically validated the feasibility of microfluidic motors through the detection of proteins with different concentrations. The change rate of the average surface concentration on the blade surface increased linearly with the rise in protein concentrations. In addition, we investigated the modulation effect of potential differences between driving electrodes on the detection performance of microfluidic motors by numerical simulations. The concentration of the adsorbed proteins on the blade surface witnessed a drop trend with the increasing potential drops because the proteins had more time to contact and adsorb on the blade surface than under small potential drops. These microfluidic motors hold good potential for environmental detection and disease diagnosis for the advantages of a low cost and gentle condition.

## Figures and Tables

**Table 1:** Comparison between this microfluidic motor and existing methods in terms of protein detection.

Method	Detection Limit	Detection Time	References
Electrochemical	~50–100 pM	~1 h	[44]
Magnetic Bead Immunocapture	~25–800 pM	~30 min	[45]
Ion Concentration Polarization	<40 pM	~20–60 min	[46]
This work	<1 pM (Theoretical)	~30 s	--

## Data Availability

The data presented in this study are available on request from the corresponding author.
